# Local Translation in Primary Afferent Fibers Regulates Nociception

**DOI:** 10.1371/journal.pone.0001961

**Published:** 2008-04-09

**Authors:** Lydia Jiménez-Díaz, Sandrine M. Géranton, Gayle M. Passmore, J. Lianne Leith, Amy S. Fisher, Laura Berliocchi, Anantha K. Sivasubramaniam, Anne Sheasby, Bridget M. Lumb, Stephen P. Hunt

**Affiliations:** 1 Department of Anatomy and Developmental Biology, University College London, London, United Kingdom; 2 Department of Pharmacology, University College London, London, United Kingdom; 3 Department of Physiology, University of Bristol, Bristol, United Kingdom; 4 Departmento Fisiología, Facultad Medicina, Instituto Neurociencias Castilla y León, Universidad de Salamanca, Salamanca, Spain; 5 IRRCS C. Mondino, Center of Experimental Neurobiology Mondino-Tor Vergata, Rome, Italy; University of Sydney, Australia

## Abstract

Recent studies have demonstrated the importance of local protein synthesis for neuronal plasticity. In particular, local mRNA translation through the mammalian target of rapamycin (mTOR) has been shown to play a key role in regulating dendrite excitability and modulating long-term synaptic plasticity associated with learning and memory. There is also increased evidence to suggest that intact adult mammalian axons have a functional requirement for local protein synthesis *in vivo*. Here we show that the translational machinery is present in some myelinated sensory fibers and that active mTOR-dependent pathways participate in maintaining the sensitivity of a subpopulation of fast-conducting nociceptors *in vivo*. Phosphorylated mTOR together with other downstream components of the translational machinery were localized to a subset of myelinated sensory fibers in rat cutaneous tissue. We then showed with electromyographic studies that the mTOR inhibitor rapamycin reduced the sensitivity of a population of myelinated nociceptors known to be important for the increased mechanical sensitivity that follows injury. Behavioural studies confirmed that local treatment with rapamycin significantly attenuated persistent pain that follows tissue injury, but not acute pain. Specifically, we found that rapamycin blunted the heightened response to mechanical stimulation that develops around a site of injury and reduced the long-term mechanical hypersensitivity that follows partial peripheral nerve damage - a widely used model of chronic pain. Our results show that the sensitivity of a subset of sensory fibers is maintained by ongoing mTOR-mediated local protein synthesis and uncover a novel target for the control of long-term pain states.

## Introduction

There is a growing awareness that local protein synthesis in dendrites and axons plays a critical role in the modulation of long-term synaptic plasticity and axon guidance during development [1]–[3]. One of the most convincing examples of a role for local translation of mRNA in axons comes from *in vitro* studies of the invertebrate Aplysia where synapse-specific facilitation requires local protein synthesis at the activated synapse to stabilise the long-term facilitation induced by application of serotonin [4], [5]. It has been argued that adjustments to local conditions at the axon terminal or region of axonal trauma would be greatly enhanced by local protein synthesis, particularly in primary afferent sensory fibers and motoneurons where the cell body can be located at a considerable distance from the axon terminals. However, local translation in mature vertebrate axons has remained controversial, primarily because of the difficulty of identifying ribosomes and the associated translational machinery *in vivo* [2]. However recent biochemical and immunohistochemical developments have begun to provide evidence that mRNA, ribosomes and other elements required for local protein synthesis can be found in mature mammalian peripheral axons [6]–[11]. For instance, the RNA binding and transport proteins Staufen and Fragile X Mental Retardation Protein have been shown to be expressed by rat primary afferent neurons and localized to peripheral and central axons [12]. It has also been shown that retrograde signal from peripheral axonal damage requires translation of vimentin and β-importin mRNAs pre-existing at the site of injury [13], [14]. Moreover, functional studies in Aplysia revealed a key role for axonal translation in injury- or depolarization-induced hyperexcitability of Aplysia sensory axons [15], [16]. Finally, a new line of evidence for the presence of mRNA in mammalian axons came from RNA-induced silencing complex studies that showed that RNA interference is functional in peripheral mammalian axons, independently from neuronal cell body or Schwann cells [17].

There is compelling evidence to implicate the mammalian target of rapamycin (mTOR), a regulator of protein synthesis, in the control of local translation of mRNA in developing axons and in dendrites *in vitro* [1], [2], [18]–[21]. mTOR together with its binding partner raptor controls translation *via* phosphorylation of both i) the eukaryotic initiation factor 4E (eIF4E)-binding protein 1/2 (4E-BP1/2) and ii) p70S6 kinase (S6K) which activates a number of downstream targets involved in translation. mTOR signalling can be inhibited by rapamycin thus preventing the phosphorylation of both S6K and 4E-BP1/2 [22]–[24].

In the present study, we show that mTOR and the related machinery for mRNA translation is present in a subpopulation of primary afferent sensory fibers in the rat skin. Primary afferents fall into two broad categories: myelinated A- fibers that signal noxious or innocuous stimuli and unmyelinated C- fibers that in rat are largely nociceptors. A- nociceptors mediate ‘first’ pain perceived as rapid and sharp and C- fibers signal ‘second’ pain, delayed, diffuse and dull [25]. Here we show that mTOR and other components of the translational apparatus are present and active under basal conditions in some A- fibers and that the response to noxious mechanical and thermal stimulation is regulated in a subset of sensory fibers by ongoing mTOR-mediated local protein synthesis.

## Results

### mTOR and other components of local protein synthesis machinery are present in subsets of myelinated primary afferent fibers in the skin

**Figure 1:**
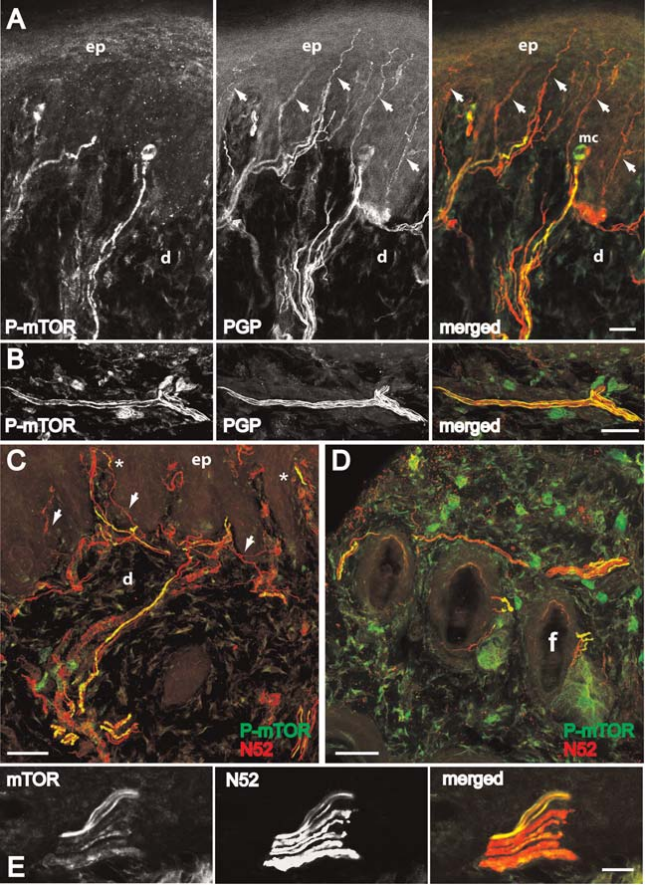
Distribution of mTOR immunoreactivity in peripheral sensory nerve fibers in the skin. Confocal images of 40 µM thick frozen sections cut perpendicular to the skin surface of the rat hindpaw. *A*, *B*, Colocalization of phospho-mTOR (*green*) and nerve fibers marker PGP (*red*), a general marker of nerve fibers, in footpad of glabrous skin. Arrows indicate PGP- positive fibers not double-labelled. *C*, *D*, Colocalization of phospho-mTOR (*green*) and myelinated fiber marker N52 (*red*) in footpad of glabrous skin *(C)* and hairy skin *(D)*. Arrows in *C* indicate N52- positive fibers not double-labelled. Asterisks (*) indicate double-labelled fibers in the area of Meissner corpuscles (sensory receptors within dermal papillae). *E*, Colocalization of phospho-mTOR and N52 in the dermis of the glabrous skin. In *A*, *B* and *E* the single staining for each antibody and the merged image are shown from left to right. In *A-E* double staining appears in *yellow*. mc, Meissner corpuscles; ep, epidermis; d, dermis; f, follicle. *A*, *E*, single focal planes; *B-D*, merge of 22–24 z-focal planes (20–21 µm depth). Scale bars, *A-E*: 50 µm.

Immunohistochemical staining of skin sections from glabrous and adjacent hairy skin of adult rat hindpaw showed that mTOR and phospho-mTOR were extensively expressed in subsets of primary afferent sensory fibers, identified from co-staining with PGP, a general marker for sensory afferents, as well as in non-neuronal cells of surrounding dermal tissue (Fig. 1A, B). These fibers never co-expressed tyrosine hydroxylase suggesting that they were not sympathetic axons (data not shown). Phospho-mTOR labelling within axons was generally continuous and we were able to trace phospho-mTOR positive axons for hundreds of microns within the dermis (Fig. 1A-D). Skin innervation by cutaneous sensory neurons has been well characterised. While C- fibers mainly terminate in different epidermal layers, A- fibers end predominantly in the dermis [26]–[28]. In the present study, we found that fibers double labelled with PGP and mTOR did not penetrate the dermal-epidermal junction (Fig. 1A). This strongly suggests that mTOR was not present in unmyelinated C- fibers which often penetrate the epidermis.

Since PGP stains all types of sensory fibers, we co-stained with N52, a specific marker for myelinated fibers. All phospho-mTOR and mTOR staining was found to co-exist with N52 immunoreactivity (a marker for the phosphorylated and non phosphorylated heavy chain of neurofilament) confirming that mTOR was largely restricted to myelinated A- fibers (Fig. 1C-E). We were able to detect mTOR staining in axon profiles ranging from 4 µm to less than 1 µm diameter (Fig. 1A-E). Counts of phospho-mTOR positive fibers from glabrous skin indicated that 35.3±1.9 % of N52- positive fibers contained phospho-mTOR (N = 3 animals, 50–100 N52 positive fibers/animal).

**Figure 2 pone-0001961-g002:**
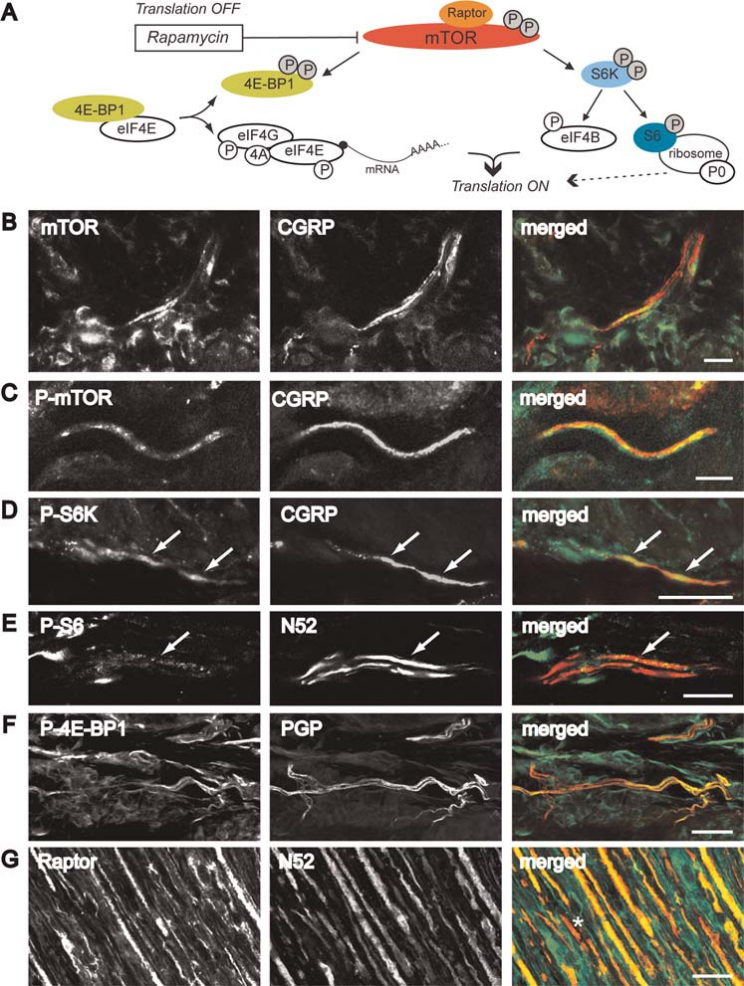
Translation-related factors are localized in peripheral nerve fibers and terminals in the skin. *A*, Diagram of mTOR signalling pathways and translational modulation. P, phosphorylation [24], [33], [34], [80]. *B-F*, Confocal images of 40 µM thick sections cut perpendicular to the plantar skin surface of the rat hindpaw. *B-D*, Colocalization of mTOR *(B)* phospho-mTOR *(C) or* phospho-S6K *(D)* and CGRP positive fibers in the dermal glabrous skin. *E*, Colocalization of phospho-S6 and N52 in the dermis of glabrous skin. *F*, Colocalization of phospho-4E-BP1/2 and PGP in the dermal glabrous skin. *G*, Colocalization of raptor and N52 in the sciatic nerve. In *B-G*, the single staining for each antibody and the merged image are shown from left to right. Double staining appears in *yellow.* White arrows indicate double-labelled fibers. Asterisk (*) indicates N52- positive fibers not double labelled. *B*, *E* single focal planes; *C*, 8 z-focal planes (2.3 µm); *D*, merge of 14 z-focal planes (8.5 µm depth); *F*, merge of 59 z-focal planes (17 µm depth); *G*, 28 z-focal planes (11 µm). Scale bars, B-E, G: 25 µm; F: 10 µm.

Some A- nociceptors have been shown to contain calcitonin gene-related peptide (CGRP) [29], [30]. We therefore co-stained sections of skin with phospho-mTOR and CGRP and found that a small number (3–5%) of phospho-mTOR positive fibers contained CGRP (N = 3 animals, 50–100 CGRP positive fibers/animal) (Fig. 2B, C).

**Figure 3:**
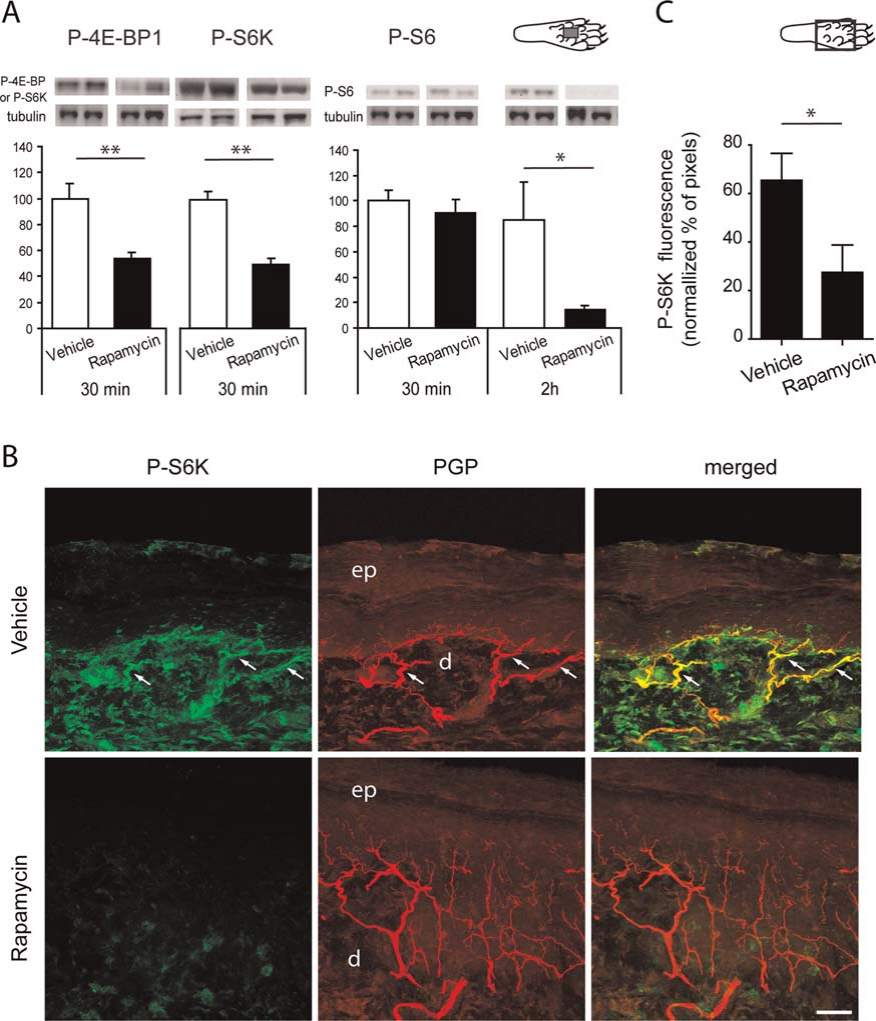
Rapamycin decreases phosphorylation of downstream targets of mTOR in the skin. *A*, Immunoblots probed with anti-phospho-4E-BP1/2, anti-phospho-S6K and anti-phospho-S6 antibodies after gel electrophoresis of lysates from skin tissue of the plantar surface of the hindpaw. Animals received an injection of rapamycin or vehicle in the center of the plantar surface 30 min or 2 h before sacrifice. The intensity of the bands for each antibody was normalized with the intensity of the β3-tubulin signal. There was a significant reduction in 4E-BP1/2 and S6K phosphorylation 30 min after rapamycin injection. The reduction in S6 phosphorylation was seen 2 h after rapamycin injection. n = 3–4 in each condition. *B*, Confocal images of phospho-S6K immunostaining (*green*) in the glabrous skin 30 min after intraplantar injection of rapamycin or vehicle in the center of the plantar surface of the hindpaw. PGP staining is shown in *red* and double staining appears in *yellow.* White arrows indicate double-labelled fibers. Note the overall decrease of phospho-S6K labelling after rapamycin injection. ep, epidermis; d, dermis. Merge of 24 z-focal planes (22 µm depth). Scale bar, 50 µm. *C*, Semi-quantification of phospho-S6K immunofluorescence in the plantar skin nerve fibers. The *y* axis represents the percentage of normalised phospho-S6K pixels above a threshold of 50. Measurements were made from confocal images as in *B*. Diagrams of the plantar surface of the rat paw indicating the area sampled (rectangle) are also included. * *P*<0.05; ** *P*<0.01.

We were also able to localize phosphorylated downstream targets of mTOR (Fig. 2A) co-existing with PGP, CGRP and N52 such as phospho-4E-BP1/2, phospho-S6K and phospho-S6 protein in skin tissue, (Fig. 2D-F and Fig. 3B). Phospho-S6K was extensively expressed in approximately 42±2.1 % of N52- positive fibers (N = 3 animals, 50–100 fibers/animal). In sections of the sciatic nerve, immunoreactivity for the mTOR co-factor raptor [31], was found extensively within the majority of N52- positive fibers but also in a small number of non-N52 positive fibers (Fig. 2G). The fact that downstream targets of mTOR were phosphorylated suggested that active translation of mRNA was occurring without external stimulation.

### The mTOR inhibitor rapamycin blocks the phosphorylation of 4E-BP1/2, S6K and S6 in skin

Activity of mTOR in the skin was assessed by quantifying phosphorylation of direct downstream targets 4E-BP1/2 and S6K. The level of phosphorylation of S6 protein was also used as a measure of S6K activity [23], [32]–[34].

Animals received intraplantar injections of the mTOR inhibitor rapamycin (50 µl of 250 µM, *i.e.* 12.5 µg) or vehicle in the center of the hindpaw. Injection of vehicle or rapamycin resulted in a mild inflammatory response at the site of injection. Using western blotting and immunohistochemistry, we found that intraplantar injections of rapamycin reduced phosphorylation of downstream targets of mTOR, therefore suggesting that mTOR activity was inhibited by rapamycin. Specifically, western blot analysis showed a significant decrease in 4E-BP1/2 and S6K phosphorylation in the skin 30 min after injection of rapamycin compared to vehicle (Fig. 3A, *P*<0.01 for both proteins). There was a significant decrease in S6 phosphorylation in the rapamycin-treated group when compared to vehicle at 2 h after treatment but not at 30 min (Fig. 3A, drug effect: F_1,8_ = 6.15, *P*<0.05, time effect: F_1.8_ = 8.08, *P*<0.05).

Immunohistochemistry and confocal analysis of phospho-S6K were also used to show that the reduced phosphorylation caused by rapamycin injections occurred within cutaneous primary afferents, as well as in the surrounding cutaneous tissue (Fig. 3 B,C). A marked decrease in S6K phosphorylation was observed 30 min after rapamycin injection throughout the treated dermis, including nerve fibers, when compared to vehicle treatment. Image analysis of immunofluorescence showed that this decrease was statistically significant when measured in nerve fibers only (*P*<0.05, N = 4 in each group) (Fig. 3C).

### Acute nociceptive thresholds are not influenced by local rapamycin injections

**Figure 4:**
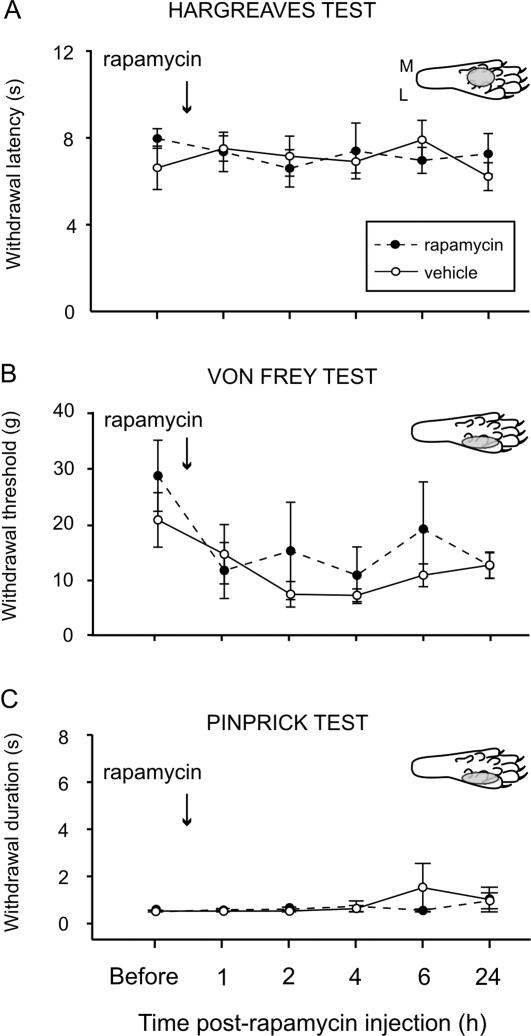
Rapamycin does not alter acute pain. *A*, *B*, *C*, Effects of intraplantar injection of rapamycin (or vehicle) in naïve rats on withdrawal latency to heat using the Hargreaves test (*A*), mechanical withdrawal threshold measured using Von Frey hairs (*B*), and withdrawal response duration after nociceptive mechanical stimulation (pinprick stimulus) of the plantar surface of the paw (*C*). See inset diagrams of the plantar surface of the rat paw for injected areas (gray circles). Mean±SEM is illustrated. M, medial; L, lateral.

Thermal and mechanical thresholds were monitored 1–24 h after local rapamycin injections into the dorsal or plantar surface of the hind paw and found to be reduced due to the local inflammation produced by the vehicle (Fig. 4 and Text S1). Rapamycin did not attenuate this inflammatory hyperalgesia and there was no difference between the vehicle and rapamycin treated animals at any time point. Given the relatively small number of fibers containing the biochemical apparatus for local translation this was not surprising.

We therefore designed a number of experiments using electromyography, behavioural analysis and the skin-nerve preparation to explore the response of subsets of nociceptors. First, we chose to use an electromyographic method that has been shown to record the separate responses of A- and C- fiber nociceptors following differential activation by heat ramps [35]. Second, we used a behavioural approach evoking primary and secondary hyperalgesia in the hindpaw by capsaicin injection, a model of injury-induced persistent pain. Capsaicin induced primary sensitization is thought to be driven by C- nociceptors and a sub-population of Aδ- nociceptors. Secondary hyperalgesia is an increased sensitivity to noxious mechanical stimulation that develops in the uninjured area of the skin (areas unstimulated by capsaicin) and is thought to be a reflection of the increased response of spinal neurons (sensitisation) to A- nociceptor stimulation [36], [37]. Finally, to directly examine the response of individual primary afferent sensory fibers, we used the skin nerve preparation [38], [39].

### Rapamycin injection into the dorsal skin of the hindpaw decreases thermal sensitivity of a subset of A- nociceptors: electromyographic studies

**Figure 5:**
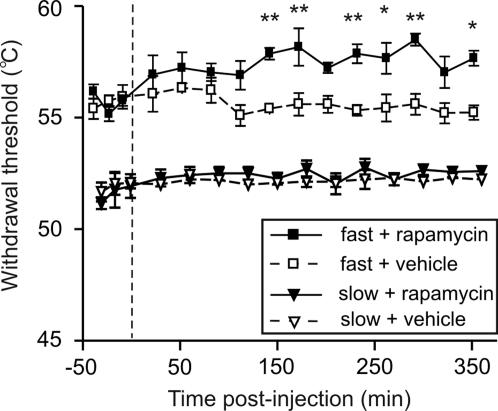
Rapamycin increases A- but not C- nociceptor-evoked paw withdrawal thresholds. Time-course effects of rapamycin or appropriate vehicle, on paw withdrawal thresholds to fast and slow heat ramps that preferentially activate A- and C- nociceptors respectively. N = 3 in each group. Mean±SEM heat withdrawal threshold (°C) for the injected hindpaw is illustrated in each panel. Vertical dashed line indicates the drug injection time. * *P*<0.05; ** *P*<0.01.

Heat-responsive and capsaicin-insensitive A- nociceptors located within the dorsal hairy skin of the hindpaw are preferentially activated by a fast heat ramp, whereas C- fibers respond only to a slower heat ramp [35], [40]. Subcutaneous injection of rapamycin into the dorsal skin of the hindpaw significantly increased threshold temperatures for paw withdrawal evoked by fast heat ramps (activating A- fiber nociceptors) from 150 min post-injection, compared to control injections of appropriate vehicle (Fig. 5; *P*<0.05, Bonferroni post-hoc test; supplementary Fig. S1B). In contrast, paw withdrawal thresholds to slow heat ramps (activating C- fiber nociceptors) remained unchanged after rapamycin (Fig. 5 and Fig. S1B).

Anisomycin (a global protein synthesis inhibitor) was used to confirm that the effects of rapamycin observed here were due to inhibition of translation. Subcutaneous injection of anisomycin also decreased thermal sensitivity after fast heat ramps (Fig. S1A; *P*<0.05, Bonferroni post-hoc test; Fig. S1C) but not slow heat ramps when compared with vehicle.

In summary, we showed that A- fiber but not C- fiber responses were attenuated by local administration of rapamycin. Furthermore, the capsaicin-insensitive A- fibers analysed were a subpopulation previously associated with the mechanical hyperalgesia that follows peripheral damage. We therefore designed experiments to test the effects of rapamycin on 1) C- fiber-induced thermal hyperalgesia and c-Fos expression and 2) A- fiber-mediated mechanical secondary hyperalgesia that develops around the site of injury.

### Intraplantar injection of rapamycin does not alter primary thermal hyperalgesia

To confirm the apparent lack of effect of rapamycin on the thermal response of C- nociceptors, we directly measured the effects of rapamycin on the development of thermal primary hyperalgesia that follows capsaicin injections into the paw. We used the results from the electromyographic experiments described above and pilot behavioural experiments as a guide to the most suitable time for behavioural testing after rapamycin injections. Rapamycin was therefore given 4 h before subsequent treatments.

**Figure 6:**
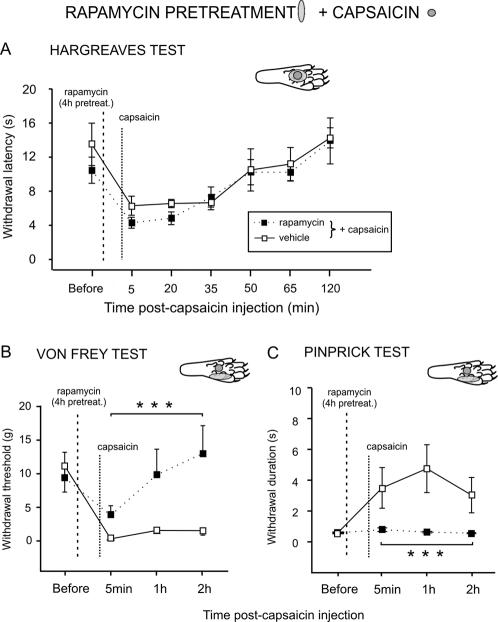
Rapamycin blocks capsaicin-induced secondary mechanical hyperalgesia but not primary hyperalgesia. *A*, Effect of rapamycin or vehicle on withdrawal latency to heat after capsaicin. Rapamycin or vehicle were injected intraplantar 4 h before capsaicin administration. All injections were made in the center of the plantar surface of the paw. *B*, *C*, Secondary mechanical hyperalgesia in the lateral plantar hindpaw was generated by injecting capsaicin into the central plantar surface of the hindpaw. Rapamycin or vehicle pre-treatment was delivered intraplantar in the lateral part of the surface of the hindpaw 4 h before capsaicin injections (see inset diagrams). *B*, Effect of lateral intraplantar injection of rapamycin or vehicle on mechanical withdrawal threshold on central plantar injection of capsaicin using Von Frey hairs. *C*, Effect of rapamycin or vehicle on withdrawal response duration to pinprick stimulation after capsaicin. Mean±SEM for the injected (left) hindpaw is illustrated in each panel. M, medial; L, lateral. *** *P*<0.001.

Capsaicin on its own increased thermal sensitivity (*i.e.* induced primary hyperalgesia) for up to 35 min after intraplantar injection (Fig. S2A). No significant changes in withdrawal latency were seen in the contralateral hindpaw (data not shown). Intraplantar pre-treatment with rapamycin or vehicle 4 h before capsaicin did not change the increased thermal sensitivity that follows capsaicin injection (Fig. 6A).

The effects of rapamycin on the development of central sensitization following capsaicin injection were also studied with c-Fos immunohistochemistry. Fos expression has been widely used to map neuronal activity within nociceptive pathways [41], [42]. We found that rapamycin did not reduce the number of Fos-IR neurons seen in the dorsal horn after capsaicin injection in the hindpaw (Text S2).

Taken together, our results strongly imply that the development of primary hyperalgesia is not sensitive to rapamycin.

### Rapamycin blocks secondary mechanical hyperalgesia induced by capsaicin

Capsaicin-insensitive A- fiber nociceptors are thought to mediate punctate secondary mechanical hyperalgesia, that is the mechanical sensitivity that develops around a site of injury [36]. Therefore, we next examined the effect of rapamycin on secondary mechanical hyperalgesia. As described above, we first induced central sensitization with an injection of capsaicin into the central part of the hind paw. Following this, we tested the mechanical sensitivity that develops around the site of injection.

Lateral areas of the skin, unstimulated by capsaicin, were pre-treated with rapamycin to determine its effects on secondary mechanical sensitivity. To determine response thresholds, both Von Frey hairs, which cover the spectrum of both A- and C- fiber mechanical response thresholds, and pinprick tests, a more specific stimulus for A- fiber nociceptors, were used.

*Von Frey Hairs testing*: Capsaicin alone (N = 8) increased mechanical sensitivity in the area of the skin unstimulated by capsaicin for up to 2 h after subcutaneous injection (see Fig. S2B).

When animals received an injection of rapamycin 4 h before capsaicin (N = 8) in the lateral part of the surface of the hindpaw, rapamycin blocked the capsaicin induced secondary hyperalgesia from 5 min to 2 h after capsaicin (post-hoc *P*<0.001 *vs*. vehicle, Fig. 6B).

Pre-treatment with a lower dose of rapamycin (2.5 µM, N = 6) had no effect on the development of secondary hyperalgesia (Fig. S3).

Pre-treatment with the global protein synthesis inhibitor anisomycin also prevented the development of the secondary hyperalgesia due to capsaicin injection (post-hoc *P*<0.001 *vs*. vehicle pre-treatment, Fig. S4A).

*Response to pinprick*: To confirm that secondary mechanical hyperalgesia can be largely abolished by rapamycin pre-treatment we examined the response to pinprick, a more specific stimulus for A- fiber nociceptors [36]. Capsaicin alone increased withdrawal duration to the pinprick stimulus in the area of secondary hyperalgesia for up to 2 h after intraplantar injection (Figure S2C). When injected into the lateral area of the paw 4 h before capsaicin administration, rapamycin completely prevented capsaicin-induced secondary hyperalgesia (Fig. 6C) (post-hoc *P*<0.001 *vs*. vehicle pre-treatment).

Again, pre-treatment with the global protein synthesis inhibitor anisomycin prevented the development of the secondary mechanical hyperalgesia that follows capsaicin injection (drug effect F_1,10_ = 7.162, *P*<0.05; Fig. S4B), confirming that the effect of rapamycin was due to inhibition of translation.

It has been shown that rapamycin forms a complex with the immunophilin FK506-binding protein 12 (FKBP12), which then inhibits the protein kinase activity of mTOR. To confirm the specificity of the action of rapamycin on mTOR, we used ascomycin, an analog of FK506, which binds to FKBP12 but does not inhibit mTOR activity [43]. Ascomycin pre-treatment did not affect capsaicin-induced secondary hyperalgesia confirming the specificity of the action of rapamycin on mTOR (Fig. S5).

In summary, rapamycin pre-treatment significantly attenuates secondary mechanical hyperalgesia tested with either Von Frey hairs or pinprick.

### Electrophysiological analysis reveals an effect of rapamycin on responsiveness of subsets of nociceptors

Our results using electromyography and behavioural techniques had indicated that the sensitivity of a subset of A- fiber nociceptors could be modified by rapamycin treatment. Although the numbers of such fibers identified with immunohistochemistry was somewhat low, we directly examined the effect of rapamycin on the response of individual primary afferent sensory fibers using the skin nerve preparation.

A total of 148 fibers were analysed in this study. Of these, 59 units (38 Aδ- and 21 C- fibers) were recorded from animals receiving rapamycin and 89 units (59 Aδ- and 30 C- fibers) from animals receiving vehicle.

**Figure 7:**
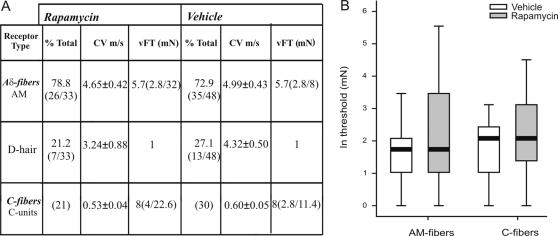
Changes in physiological properties of mechano-sensitive fibers after rapamycin treatment. *A*. Means±SEM are shown. CV, conduction velocity; vFT, Von Frey threshold. Von Frey thresholds are given as the median with the 1st and 3rd quartiles in brackets. *B*. Box plot of Von Frey thresholds for mechano-sensitive AM- and C- fibers. Data have been normalized by logarithmic (ln) transformation. Horizontal dark bars indicate the median.

The proportion of mechano-insensitive Aδ- fibers was similar for animals receiving rapamycin (13%) or vehicle (12%). The properties of mechano-sensitive fibers are summarised in Fig. 7 and Fig. S6. The incidence of low-threshold D- hair receptors and high-threshold AM- fibers was unchanged following treatment by rapamycin ([73% and 27%] and [79% and 21%] of [Aδ- and D- hair] in vehicle and rapamycin treated animals, respectively).

Rapamycin increased the mean Von Frey threshold for AM- [5.3 (3.5–8.0) mN to 8.4 (4.7–15.0) mN] and C- fibers [5.4 (3.8–7.8) mN to 8.3 (4.9–13.8) mN] by 58% and 54%, respectively (Fig. 7), indicating that rapamycin had an effect on subpopulations of both C- and Aδ- nociceptive fibers, though this did not quite reach significance (overall effect F_1,112_ = 3.4, *P* = 0.068). Although, the median of the AM- fibers was identical for animals receiving rapamycin or vehicle (5.7 mN), a larger interquartile range (IQR; 29 mN *versus* 5.2 mN in rapamycin and vehicle, respectively) and 3^rd^ quartile (32 mN *versus* 8 mN in rapamycin and vehicle, respectively) implied that the Von Frey threshold had increased in a proportion of AM- fibers after rapamycin. Whilst this is also true for C- fibers (median 8 mM), the difference in interquartile range and 3^rd^ quartile [39] between groups receiving vehicle [IQR, 8.6; 3Q, 11.4] or rapamycin [IQR, 18.6; 3Q, 22.6] was less pronounced. To determine whether the effect of rapamycin was being masked by fibers insensitive to treatment, we statistically evaluated the effect of rapamycin on AM- and C- fibers separately introducing the factor ‘median’. Results indicated that the effect of rapamycin on a subset of fibers was significant for AM- fibers (F_1,62_ = 4.7, *P* = 0.035) but not for C- fibers (F_1,52_ = 1.2, *P* = 0.279) (Fig. 7).

### Rapamycin reduces mechanical sensitivity in a rat model of chronic pain

**Figure 8:**
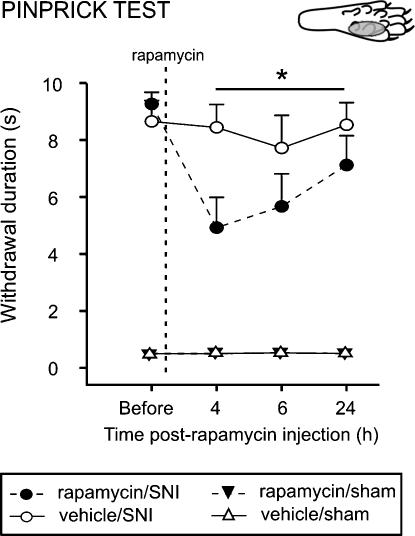
Rapamycin attenuates mechanical pinprick hyperalgesia in the spared nerve injury (SNI) model. Effect of lateral intraplantar injection of rapamycin, or its vehicle, on withdrawal response duration (s) after nociceptive mechanical stimulation (pinprick stimulus) of the lateral plantar surface of the paw of SNI animals, or sham animals. Mean±SEM is illustrated. N = 6–9 in each group. * *P*<0.05.

Finally, we extended the observation that rapamycin reduces secondary mechanical sensitivity to a model of neuropathic pain. The increased pain sensitivity in neuropathic pain models is thought to reflect, in part, maintained primary and therefore secondary mechanical hyperalgesia. Following spared nerve injury (SNI), rats showed an enhanced response to pinprick stimulation in the lateral part of the hindpaw, the sural territory, 6 days after surgery [44] (see Text S3 for details). On day 6 following surgery, animals received an intraplantar injection of 50 µl of rapamycin or vehicle in the lateral area of the hindpaw. Intraplantar rapamycin treatment resulted in a decrease in time holding the paw 4 to 24 h post-injection (F_1,16_ = 4.9; *P*<0.05; Fig. 8), with a maximum reduction of 53% seen at 4 h. With a single time point post-hoc analysis, the effects of rapamycin were significant at the 4 h time point (F_1,16_ = 6.933, *P* = 0.018). No changes in withdrawal duration were observed in sham animals after injection of rapamycin or vehicle.

## Discussion

We present evidence to show that the machinery for mTOR-mediated local mRNA translation is found in a subpopulation of myelinated sensory fibers. Furthermore, we demonstrate that local treatment with rapamycin, an inhibitor of mTOR activity, both inhibits local protein synthesis and reduces the mechanical and thermal response of A- nociceptors. We therefore propose that ongoing local translation of mRNA maintains the sensitivity of this subset of nociceptors.

### The responsiveness of A- fiber nociceptors is maintained by mTOR-dependent local translation of mRNA

In this study, we showed that acute nociceptive thresholds are uninfluenced by local rapamycin administration. Given the relatively small number of fibers containing the apparatus for local translation, this was not surprising. However, by using physiological and behavioural assays we were able to unmask a significant influence of local protein synthesis on maintaining the threshold of a subset of nociceptors. We provide here several lines of evidence to support the argument that A- fiber nociceptors have the capacity to translate mRNA locally. This supports previous studies demonstrating the presence of ribosomal particles in myelinated primary afferent sensory fibers [8], [9]. Furthermore, our data imply that local mRNA translation in A- fibers is an active process under basal conditions which maintains nociceptor sensitivity. This is also supported by recent investigations where knock down of FMRP, a RNA binding and transport protein which is found in sensory axons, was linked to deficits in mGluR-mediated peripheral nociception [45]. First, our immunohistochemical results showed that the translational machinery was present in myelinated fibers (Fig. 1). A minor subset of these mTOR positive fibers also contained CGRP (Fig. 2) which has been shown to be expressed by rapidly conducting A- fiber nociceptors [29], [46]. Second, rapamycin increased thermal thresholds only to fast thermal ramps that preferentially activate capsaicin-insensitive A- fiber nociceptors, but not to slow heat ramps that preferentially activate capsaicin-sensitive C- fiber nociceptors [35], [40], [47] (Fig. 5). This lead us to study secondary mechanical hyperalgesia which is known to be exclusively mediated by capsaicin-insensitive cutaneous A- fiber nociceptors [36], [37], [48]. Secondary hyperalgesia is characterized by increased sensitivity, particularly to punctate mechanical stimuli, in the undamaged skin that surrounds the site of injury (in this case the capsaicin injection site). Increased secondary mechanical sensitivity has been shown to be the result of amplification generated, in part, by sensitized dorsal horn neurons [49]. We confirmed that secondary hyperalgesia was substantially attenuated by local injection of rapamycin (Fig. 6). In other words, reducing the sensitivity of this subset of A- nociceptors peripherally with local administration of rapamycin diminished the input to the dorsal horn and thus the subsequent central amplification of the A- fiber response. In models of neuropathic pain such as SNI, heightened sensitivity to mechanical stimulation is also thought to be the result of amplification by central sensitization [50]–[52] and we found it was also reduced by rapamycin (Fig. 8). SNI- induced mechanical hyperalgesia was also found reduced in FMRP-KO mice [45]. Finally, direct measurements of A- fiber sensitivity revealed a shift to higher mechanical thresholds following rapamycin treatment (Fig. 7).

### Effect of rapamycin on other types of sensory fibers

Several lines of evidence suggested that C- fibers did not have the capacity for local mTOR-dependent translation at least within cutaneous tissue. Fibers which penetrate the epidermis are mostly C- fibers and these were always negative for the markers of translational machinery used here. Capsaicin injection generates C- fiber-mediated thermal hyperalgesia and a robust expression of c-Fos in dorsal horn neurons but both of these outcomes were unchanged by prior treatment with rapamycin (Fig. 6A and Text S2). However, analysis of the mechanical thresholds of C- fibers did suggest that a subpopulation of C- fibers was somewhat influenced by rapamycin (Fig. 7) and indeed the expression of raptor overlapped with non-N52 positive profiles in the sciatic nerve. This implies that a small number of C- fibers might have the capacity for mTOR mediated local protein synthesis but went undetected in our immunohistochemical or behavioural assays. This may be a sensitivity issue or because this population terminated deep within the dermis and was thus not detected in our immunohistochemical analysis [53]. It is also possible that C- fibers can support a rapamycin insensitive translation mechanism, such as IRES-dependent translation. However, this has not been investigated here where our focus was entirely on rapamycin sensitive mTOR processing. The presence of mTOR positive large diameter myelinated fibers terminating in the vicinity of Meissner corpuscles - sensory receptors within dermal papillae - also suggests that low threshold Aβ- fiber sensitivity may support local translation. Aβ- fibers are generally regarded as low threshold mechanoreceptors although there is evidence that >20% may be nociceptors [29], [54].

### The control of A- nociceptor sensitivity

Our findings throw new light on the control of A- fiber sensitivity. Previously, A- fibers had not been thought to possess the inherent plasticity of C- fibers [50], [55], [56] and modulation of A- fiber sensitivity was assumed to be wholly contingent upon central sensitization established by activation of C- fibers [36], [57]. In other words, the excitability of central dorsal horn neurons increases following a C- fiber barrage and the response to subsequent A- fiber inputs is amplified by these sensitized neurons. The data presented here implies that, even in the absence of C- fibers activation, local translation of mRNA regulates the sensitivity of some A- fibers and therefore modulation of mTOR signalling can influence the response of these fibers. The thermal and mechanical sensitivity of these nociceptors suggest that they may be type I A- fiber nociceptors [58]. Local translation may regulate A- fiber sensitivity through modulation of either the transduction process or excitability of the primary afferent terminal [59], but at present the mechanism is unclear. Previous *in vitro* work on hippocampal pyramidal neuron dendrites demonstrated that activation of mTOR can relieve microRNA inhibition of translation leading to structural changes in the spine [21] or suppress potassium channel Kv1.1 expression which in turn would increase excitability [19]. However, modulation of channel expression in hippocampal neurons occurred within 75 min [19] and in our experiments A- fiber nociceptor sensitivity was not seen to change for 2–3 h after rapamycin administration (Fig. 5), implying that regulation of A- fiber excitability by rapamycin was a relatively slow process. We hypothetize that local protein synthesis is continuously replenishing proteins essential for the full response of the fiber to noxious stimulation within the skin sensory terminations. In the presence of rapamycin, the inhibition of mTOR signalling prevents the replenishment of the stores of these key proteins. From our results, 2 to 3 h is required for a significant degradation of these pools of proteins and loss of fiber sensitivity.

mTOR is known to play a crucial role in the signalling pathway that regulates cell growth in response to a variety of external stressors and cues including nutrients and growth factors, hypoxia, DNA damage and osmotic stress [60] and it seems likely that peripheral changes in the physiological state of the body, for example during illness, are reflected in modulation of A- fiber sensitivity. For example, pinprick hyperalgesia is attenuated in diabetic patients, both in those with painful neuropathy and those without symptoms [61]. This may be related to the decreased levels in diabetes of insulin and IGF1, which are both powerful activators of the mTOR pathway and which could act on the insulin-like growth factor receptor which is expressed on small and medium sized dorsal root ganglia neurons [60], [62].

One particular concern in the present series of experiments was the possible contribution of non-neuronal cells present in cutaneous tissues to maintaining the sensitivity of nociceptors through an mTOR mediated synthesis or release of trophic factors. However, while it is difficult to completely rule out an effect of rapamycin on supporting cells, we believe it to be unlikely for several reasons. First, inflammatory or primary hyperalgesia produced by vehicle injection and known to be due to the release of a large number of factors from damaged tissue [58], was not reduced by rapamycin injection. Second, non-neuronal tissue influences would have had to specifically influence subsets of A- fibers. It has indeed been shown that growth factors, such as brain-derived neurotrophic factor (BDNF), released under basal conditions, maintain the sensitivity of myelinated mechanonociceptors [63]. However, in our capsaicin model, injection of the trkB-IgG fusion protein (an inhibitor of BDNF function [64]) into the area of secondary hyperalgesia (10 µg/50 ul, 4 h prior to capsaicin injection) had no effect on pinprick pain thresholds compared to vehicle controls (data not shown).

### Local translation of mRNA and the central process of primary afferents

The evidence presented here for local translation of mRNA in myelinated axons innervating cutaneous tissue also raises the possibility that a similar process is operating at the sites of termination of sensory afferents within the dorsal horn. In most of the studied cases, the transport of molecules from the dorsal root ganglion is in both directions, to the periphery and along the central process that terminates within the superficial dorsal horn. It therefore seems highly likely that local translation of mRNA occurs in central terminals of A- fiber nociceptors. In Aplysia, neurotransmitter modulation of local translation of mRNA in sensory afferents leads to long-term changes in synaptic efficacy [4], [5]. A similar relationship may exist in the mammalian spinal cord, the activity of central neurons and non-neuronal cells modulating local translation in primary afferents and generating long term changes in the efficacy of sensory transmission through activation of receptors such as mGluR5 [43], [65]–[68] or the 5HT3 receptor [69], [70].

In summary, in the present study, we show that on-going mTOR-mediated local protein synthesis in cutaneous A- fiber nociceptors regulates pain sensitivity and reveal a novel route for the control of pain.

## Materials and Methods

### Subjects

All procedures complied with the UK Animals (Scientific Procedures) Act 1986. Male Sprague Dawley rats (170–200 g; Central Biological Services, University College London, UK) were used except for electromyographic (EMG) studies when male Wistar rats (280–310 g; University of Bristol, UK) were used. Animals were kept in their home cages at 21°C and 55% relative humidity with a light-dark cycle of 12 h (lights on at 08:00 h). Food and water were provided *ad libitum.* All efforts were made to minimise animal suffering and to reduce the number of animals used. A total of 300 animals were used for the study.

### Antibodies and drugs

Anti-phospho-mTOR (Ser2448; used at a concentration of 1∶1000 for immunohistochemistry; Cat. No.: 2971), anti-mTOR (1∶1000; Cat. No.: 2972), anti-phospho-4E-BP1/2 (Thr37/46; 1∶1000; Cat. No.: 9459), anti-phospho-S6 (Ser235/236; 1∶1000; Cat. No.: 2211), anti-phospho-S6K (Thr389; 1∶2000; Cat. No.: 9206) and anti-raptor (1∶5000; Cat. No.: 2280) antibodies were obtained from Cell Signaling Technology (MA, US). Anti-calcitonin gene related peptide antibody (CGRP; 1∶2000, Cat. No.: AB5920) was obtained from Chemicon (CA, US). The anti-protein gene product 9.5 antibody (PGP; 1∶500; Cat. No.: RA95101 was obtained from Ultraclone (Cambridge, UK). The antibody to mouse anti-neurofilament 200 kDa clone N52 (N52; 1∶2000; Cat. No.: N0142) was obtained from Sigma (Pool, UK). Finally, anti-tyrosine hydroxylase antibody (1∶5000; Cat. No.: 657014) was from Calbiochem (Darmstadt, Germany). N-Vanillylnonanamide (synthetic capsaicin) and anysomicin were purchased from Sigma, rapamycin from LC Laboratories (MA, US) and ascomycin from Alomone Labs (Jerusalem, Israel).

### Intraplantar injections of capsaicin

N-Vanillylnonanamide (synthetic capsaicin) solution was prepared at a concentration of 10 mM and made up in a vehicle of saline containing 10% ethanol and 10% Tween 80. All injections were given in a volume of 10 µl. To prepare for the injection, rats were gently wrapped in a cotton towel with the left hindpaw exposed. During the injection the needle penetrated the skin just distal to the targeted area which was the center of the plantar surface of the left hindpaw (see Fig. S5B). Care was taken to deliver each injection superficially into the skin. Injection of capsaicin, but not of the vehicle, produced immediate “nocifensive” behaviour (lifting, licking and/or shaking of the paw) that lasted 1–3 min.

### Intraplantar injections of rapamycin/anisomycin/ascomycin

Rapamycin was prepared in solutions of 2.5 µM and 250 µM in a vehicle of saline containing 0.2% or 20% ethanol respectively. Rapamycin was always given at a concentration of 250 µM unless otherwise stated. Anisomycin was prepared at a concentration of 4.7 mM [71], [72] in a vehicle of saline containing 0.2% ethanol. Ascomycin was prepared at a concentration of 250 µM in a vehicle of saline containing 20% ethanol [43]. All injections were given in a volume of 50 µl under isoflurane (2%). Again, the needle penetrated the skin superficially, just distal to the targeted area which, depending on the experiment, was either the lateral part or the center of the plantar surface of the left hindpaw. In experiments involving EMG recordings and in the skin nerve preparation, the injections were delivered in the dorsal surface of the paw. Injections of rapamycin/anisomycin/ascomycin or vehicle did not produce any impairment in locomotion or guarding behaviour.

### Immunocytochemistry

For immunohistochemistry, rats were deeply anaesthetized with pentobarbital after injections of 250 µM rapamycin or its appropriate vehicle in the center of the plantar surface, and perfused transcardially briefly with saline containing 5 000 I.U./ml heparin followed by 4 % paraformaldehyde (PFA) in 0.1 M phosphate buffer (PB) containing 0.05 M sodium fluoride (250 ml per rat). The glabrous skin of the hindpaw was dissected out around the foot pads (see diagram of the paw in Fig. 3C), post-fixed in the same PFA solution for 2 h and transferred into a 30 % sucrose solution in PB containing 0.01 % azide, for a minimum of 24 h. Tissue was cut perpendicular to the surface of the skin on a freezing microtome at 40 µm. A similar protocol was used for sciatic nerve. All primary antibodies but anti-tyrosine hydroxylase followed a same protocol with a tyramide signal amplification step. Sections were left to incubate with primary antibodies for 3 days at 4°C. Appropriate biotinylated secondary antibodies were used at a concentration of 1∶400 for 90 min. Samples were then incubated with avidin biotin complex (ABC Elite, Vector Lab., CA, US) (1∶250 Vectastain A+1∶250 Vectastain B) for 30 min followed by a signal amplification step with biotinylated tyramide solution (1∶75 for 7 min; Perkin Elmer, MA, US). Finally, sections were incubated with FITC-avidin for 2 h (1∶600). For anti-tyrosine hydroxylase antibody, sections were incubated with primary antibody for 3 days at 4°C. Then the biotinylated secondary antibody (1∶500) was left on for 2 h. Finally, sections were incubated with strepavidin-Alexa 488 (1∶500, Invitrogen, CA, US) for 2 h. For double labelling, stained sections were left for 24 h at room temperature with N52, PGP or CGRP as described above. Appropriate direct secondary was applied at a concentration of 1∶500 and incubated for 2 h. All sections were coverslipped with Gel Mount Aqueous Mounting Medium (Sigma) to protect the fluorescence from fading and stored in dark boxes at 4°C. Controls included omission of the first or second primary antibodies or addition of blocking peptides when available (phospho-mTOR and phospho-S6). We also confirmed antibody specificity by western blot. Single or double bands of appropriate molecular weight were found for mTOR, phospho-mTOR, phospho-S6K, phospho-4E-BP1/2 and phospho-S6.

### Image analysis and quantification of immunofluorescence

All images of double stained skin tissue were acquired by confocal microscopy using a laser scanning microscope (Leica TCS NT SP). Sequential laser channel acquisition was used to prevent generating false positives by ‘bleed through’ of immunofluorescence from one channel to the other. Images were obtained primarily by merging 8–59 z-focal planes, to try capture as much of a nerve fiber or fiber bundle as possible, or single focal plane acquisition, as stated (Fig. 1–3). Since skin is a complex tissue and non-neuronal cells express translational machinery, when z series were acquired, we examined single focal planes within the stack to eliminate false positives due to inadvertent inclusion of non-neuronal tissue overlapping positive axonal staining. For fiber counting, we counted the number of double labelled fibers in a total of 50–100 N52- positive fibers per animal (total of 3 animals) under confocal microscopy.

For the semi-quantitative analysis of phospho-S6K immunofluorescence, equal staining in both treatment groups was insured by processing all tissue in parallel, from animal perfusion to tissue staining. The signal in the PGP stack was thresholded and used to create a volume mask for the bright PGP- positive fibers. The phospho-S6K z-series was then applied onto this mask and the pixel intensities normalised to the volume of the PGP-positive fibers [73]. The frequency of distribution of the normalised phospho-S6K pixel intensities was expressed as percentage of pixels above the arbitrary value of 50. One to three sections were analysed for 4 animals per group (rapamycin *vs* vehicle). Image analysis was performed with the NIH software Image J (1.34s). Post-acquisition processing was performed with Adobe Photoshop and Adobe Illustrator.

### Tissue collection and immunoblotting

For fresh tissue collection, animals were terminally anaesthetized with CO_2_ 30 min or 2 h after injections of 250 µM rapamycin or its appropriate vehicle in the center of the plantar surface. A 0.5 cm × 0.5 cm square of skin tissue (see diagram of the paw in Fig. 3A) was dissected out from the ventral surface of the hindpaw around the injection site, making sure to avoid the foot pads. Samples were then stored at −80°C until further processing. For protein extraction, one sample of skin tissue was homogenized in 500 µl of lysis buffer (1% Np-40, 20 mM Hepes pH 7.4, 100 mM NaCl, 100 mM NaF, 1 mM Na_3_VO_4_, 5 mM EDTA with 1× protease inhibitor cocktail (Sigma); 1× phosphatase inhibitor cocktail I and II (Sigma)) and incubated on ice for 2 h. Samples were then centrifuged at 13 000 rpm for 15 min and supernatants collected. Total protein concentration was assessed using a bicinchoninic acid (BCA) protein assay kit (Pierce Biotechnology, IL, US) before each preparation of protein samples. Samples (10 µg of proteins per well) were run on 8 % or 10 % Bis-Tris gels (Biorad Laboratories, CA, US) for detection of phospho-4E-BP1/2 or phospho-S6K and phospho-S6 respectively. Proteins were transferred onto a PVDF membrane (Biorad). Membranes were blocked in 10 mM Tris-HCl pH = 7.5, 150 mM NaCl, 0.05 % Tween 20 (Sigma) and 0.24 % I-Block (Tropix, MA, US) and incubated with phospho-4E-BP1/2, phospho-S6 or phospho-S6K antibody (Cell Signaling, 1∶1000, overnight at 4°C). After washes, an appropriate HRP-conjugated secondary antibody was applied for 45 min. HRP activity was visualized by applying a chemiluminescent substrate (ECL; Amersham Pharmacia Biotech, NJ, US) and using Chemi Doc XRS from Biorad. Membranes were then washed and incubated with β3-tubulin antibody (1∶2000; Promega, WI, US) for 45 min, and further processed as described above. Signal intensity was measured using Quantity One software (Biorad). For each sample and each membrane signal for phospho-4E-BP1/2, phospho-S6 and phospho-S6K was normalized with the intensity of the corresponding β3-tubulin signal. For each condition a minimum of 3 replicates from 3 different animals were run. The mean value obtained for the vehicle treatment at 30 min was arbitrarily set at 100%. For an extra confirmation of equal loading of proteins, membranes were stained with Coomassie dye and staining of the wells visually compared.

### Electrophysiology

#### Electromyographic dissociation of A- and C- fiber responses

Recording of electromyographic (EMG) activity was performed as described by McMullan *et al.* [35]. Cannulation of the external jugular vein (for maintenance of anaesthesia), carotid artery (to monitor arterial blood pressure) and trachea (for regulation of breathing) was performed under halothane (4%) in oxygen. Maintenance of anaesthesia, was achieved by constant i.v. infusion of alphaxalone/alphadolone (Saffan, Schering Plough Animal Health, UK; 14–27 mg.kg^−1^.hr^−1^) through the external jugular vein. Recording of EMG activity was achieved using bipolar electrodes made from short lengths of Teflon-coated steel wire (0.075 mm, Advent Research Materials, Oxford, UK) inserted into the left biceps femoris. The EMG signal was amplified (x5K; Neurolog NL104A, Digitimer, UK) and filtered (50 Hz–5 KHz; Neurolog NL125, Digitimer, UK), before being captured for subsequent analysis via a 1401plus (CED, UK) onto a PC running Spike2 v5.13 software (CED, UK). Following surgery, anaesthesia was reduced to a level at which animals were moderately responsive to firm pinch of the contralateral forepaw and corneal stimulation. Animals were allowed to stabilise at this level for a minimum of 30 min. A- fiber (myelinated, capsaicin-insensitive) heat nociceptors or C- fiber (unmyelinated, capsaicin-sensitive) heat nociceptors on the dorsal surface of the hindpaw were preferentially stimulated via fast (7.5±1°C s^−1^) or slow (2.5±1°C s^−1^) rates of heating, respectively, using a constant bulb voltage as described previously [35]. The cut-off temperature of the heat lamp was controlled via a Spike2 script to prevent tissue damage. Alternating fast and slow heat ramps were carried out at 8 min intervals and threshold temperature at which the withdrawal reflex occurred recorded. Subcutaneous injection of 250 µM rapamycin, 4.7 mM anisomycin or the corresponding vehicle took place once a steady baseline of paw withdrawal thresholds had been achieved. Fast and slow heat ramps were resumed and paw withdrawal thresholds measured: a ‘pair’ of fast and slow heat ramps was carried out every 30 min, with an 8 min inter-stimulus interval, and continued for 6 h post-injection. In some experiments fast ramps alone were used.

### The skin-nerve preparation

The *in vitro* rat skin-saphenous nerve preparation was used to record single nerve fiber activity and has been described in detail elsewhere [74], [75]. All experiments were performed blind to treatment. Rapamycin or vehicle was injected subcutaneously into the dorsum of the hindpaw of adult Sprague-Dawley rats (140–200 g) 2 h prior to dissectionRats were killed by CO_2_ asphyxiation followed by cervical dislocation. The skin was excised together with the saphenous nerve trunk, mounted corium side up in an organ bath and continuously superfused (16 ml min -1) with an oxygenated modified synthetic interstitial fluid (SIF) containing (in mM): NaCl, 139; NaHCO3, 21; Glucose, 10; NaH2PO4, 0.6; KCl, 3.5; MgCl2, 1; CaCl2, 1.3 at pH7.4 and a temperature of 32°C.

The receptive fields of single fibers were identified following manual probing of the skin with a blunt glass rod and electrical stimulation of the receptive field using a Teflon-coated steel electrode (Linton Instruments, UK).

Fibers were classified according to their conduction velocity, Von Frey threshold and response to suprathreshold force (3 times threshold force). Fibers conducting below 1.2 m/s were classed as unmyelinated C- fibers and those conducting between 1.2 m/s and 10.0 m/s as thinly-myelinated Aδ- fibers.

The Von Frey threshold was determined using a series of calibrated Von Frey hairs with a uniform tip diameter of 0.8 mm and was taken as the minimum force required to elicit 3 or more action potentials. Aδ- fibers with a threshold of 1 mN and a rapidly adapting response to suprathreshold force were classed as D- hair mechanoreceptors whilst those with a threshold of ≥1 mN and a slowly adapting response to suprathreshold force were classed as high threshold Aδ- mechanonociceptors, often referred to as AM- fibers [38], [76]. Some Aδ- fibers were insensitive to mechanical stimulation and could only be identified using electrical stimulation and were therefore classed as mechano-insensitive fibers.

Single fiber recording was performed between 4 and 9 h following administration of rapamycin or vehicle. Data were analysed offline using Spike2 software (version 2.24, Cambridge Electronic Design).

### Behavioural experiments

In all experiments the observer was not aware of the substance and/or dose given in the intraplantar injections.

### Mechanical stimulation:

*Von-Frey test.* Mechanical sensitivity was assessed using the von-Frey test based on that described by Tal and Bennett [77]. Animals were allowed to habituate to the experimental apparatus for 10-15 min before testing began. Animals were habituated over a period of 2–3 consecutive days by recording a series of baseline measurements. A series of calibrated Von-Frey hairs were applied to the lateral plantar surface of the paw, in ascending order. The threshold was taken as the lowest force required to elicit a response to one of five repetitive stimuli, with an interstimulus interval of 5 s.

*Pinprick test.* The pinprick test was performed as described by Tal and Bennett [77]. Animals were placed on an elevated wire grid and habituated over a period of 2-3 consecutive days by recording a series of baseline measurements. The point of a safety pin was applied to the lateral part of the plantar surface of the paw at an intensity sufficient to indent but not penetrate the skin. The duration of paw withdrawal was recorded with a minimum arbitrary value of 0.5 s for a brief normal response and a maximum cut-off of 10 s.

*Randall-Siletto test:* The Analgesy-Meter (Randall-Selitto test) from Ugo-Basile (Italy) was used. Animals were left to habituate to the experimental room in their home cage for 15 min before the beginning of each testing session. Each animal was tested 4 times with a resting time of 10 min between each measurement. No extra discs were added to the basic settings of the apparatus, and paw withdrawal (not vocalization) was taken as a measure of pain threshold.

*Thermal stimulation.* Thermal withdrawal thresholds were determined as described by Hargreaves *et al.* [78]. Animals were allowed to habituate to the apparatus (Plantar Test Apparatus, Stoelting, IL, US) for 10–15 min before testing began. Baseline withdrawal latencies to an infrared heat stimulus were measured and recorded over a period of 3 consecutive days. Each hindpaw received 4 stimuli, alternating between paws. The inter-stimulus interval for each paw was at least 1 min. Four readings were collected from each paw. Withdrawal latencies were defined as the mean of the last three readings.

*Spared nerve injury surgery.* The spared nerve injury (SNI) was performed as described by Decosterd and Woolf [44]. Under 2 % isoflurane anaesthesia the biceps femoris muscle was exposed. A section was made through the biceps femoris to expose the sciatic nerve and its three terminal branches: the sural, common peroneal and tibial nerves. The common peroneal and tibial nerves were tightly ligated with 5.0 silk and sectioned distal to the ligation, removing 2–4 mm of the distal nerve stump. Care was taken to avoid touching or stretching the spared sural nerve. Muscle and skin were closed in two separate layers. For sham surgery, the sciatic nerve was exposed as described above but no contact was made with the nerve. Behavioural testing began the day after surgery and continued for 6 days post surgery.

*Statistical data analysis.* Repeated measures ANOVA followed by Tukey or Bonferroni post-hoc analysis where appropriate (SPSS+), was used to analyse all behavioural data, including EMG studies. The Greenhouse-Geisser ‘ε’ correction was applied to compensate for any violation of sphericity. If the Levene's test for normal distribution was significant then data were normalized by logarithmic (log) transformation. When data were analysed as percentage of baseline, care was taken to check that the baseline raw data for the different groups were not different. When the two vehicles for different concentrations of rapamycin and anisomycin were compared, there was no difference in withdrawal responses. Therefore, results obtained for all vehicle treatments were pooled for statistics and graphs. For western blots, normalised signals were compared in vehicle and rapamycin treated animals by a Student's t-test (phospho-4E-BP and phospho-S6K) or multivariate analysis followed by appropriate post-hoc tests (phospho-S6). For semi-quantification of immunofluorescence intensity, the frequency of distribution of the normalised phospho-S6K pixel intensities was compared in vehicle and rapamycin treated animals by a Student's t-test. Data are presented as mean±SEM. For all experiments, the level of significance was set at P<0.05. For the skin nerve preparation experiments, Von Frey thresholds were expressed as the geometric mean (in mN) together with their 95% confidence limits in brackets or as the median with 1^st^ and 3^rd^ quartiles. Descriptive statistics were calculated according to Tukey [79]. All statistical tests were performed in SPSS. To evaluate the effect of rapamycin on AM- and C- fibers, data were ln transformed and variances were analysed by univariate analysis with ‘treatment’ (vehicle or rapamycin) and ‘fibers’ (both AM- and C- fibres) as factors. We also included the ‘median’ as an additional factor: fibers with thresholds below or above the median were assigned the coefficient 1 or 2, respectively. For consistency, the median was assigned both coefficients in groups where there were an odd number of fibres.

## Supporting Information

Text S1 Acute nociceptive thresholds are not influenced by local rapamycin injections
(0.02 MB DOC)

Text S2 Rapamycin does not reduce the number of Fos-IR neurons in dorsal horn induced by capsaicin injection in the hindpaw
(0.03 MB DOC)

Text S3 SNI surgery
(0.02 MB DOC)

Figure S1 Effects of subcutaneously injected rapamycin or anisomycin on A and C nociceptor-evoked paw withdrawal thresholds. A, Time-course effects of anisomycin (50 µl, 4.7 mM), or vehicle, on paw withdrawal thresholds to fast and slow heat ramps that preferentially activate A- and C-nociceptors respectively. N = 3 in each group. Mean±SEM heat withdrawal threshold ({degree sign}C) for the injected hindpaw is illustrated. Vertical dashed line indicates the drug injection time. B, C, Area under the curve between 150–360 minutes post-injection of rapamycin (B) and anisomycin (C). The data are normalised with respect to the effect of vehicle injection over the same time period; data are expressed as mean±SEM and analysed using student's paired t-test. *, P<0.05; **,P<0.01.
(1.71 MB TIF)

Figure S2 Capsaicin induces local increase in thermal and mechanical sensitivity. Effects of intraplantar injection of 10 µl capsaicin (10 mM) on withdrawal latency to heat, measured in the center of the hindpaw (A), mechanical flexor reflex withdrawal threshold measured in the lateral surface of the paw (B), withdrawal response duration after nociceptive mechanical stimulation (pinprick stimulus) of the lateral plantar surface of the paw (C). Mean±SEM is illustrated in each panel.**, P<0.01; ***,P<0.001. N = 5–6 in each group.
(2.43 MB TIF)

Figure S3 Low dose of rapamycin (2.5 µM) has no effect on capsaicin-induced increased mechanical sensitivity. Secondary mechanical hyperalgesia in lateral plantar hindpaw was generated by injecting capsaicin into the central plantar surface of the hindpaw. Effect of lateral intraplantar injection of rapamycin 2.5 µM or vehicle on mechanical withdrawal threshold measured after injection of capsaicin using Von Frey hairs. N = 6 per group.
(0.85 MB TIF)

Figure S4 Anisomycin blocks capsaicin-induced secondary mechanical hyperalgesia. Secondary mechanical hyperalgesia in lateral plantar hindpaw was generated by injecting capsaicin into the central plantar surface of the hindpaw. Effect of lateral intraplantar injection of anisomycin (50 µl, 4.7 mM; 4 h before capsaicin) or vehicle on mechanical withdrawal threshold measured after injection of capsaicin using Von Frey hairs (A) and on withdrawal response duration to pinprick stimulation after capsaicin (B). (N = 7–8 for Von Frey and N = 12 for pinprick).
(1.43 MB TIF)

Figure S5 Ascomycin does not change the secondary mechanical hyperalgesia that follows capsaicin injection. Secondary mechanical hyperalgesia in lateral plantar hindpaw was generated by injecting capsaicin into the central plantar surface of the hindpaw. Effect of lateral intraplantar injection of ascomycin or vehicle on withdrawal response duration to pinprick stimulation after capsaicin. (N = 12)M
(0.74 MB TIF)

Figure S6 Dotplots showing Von Frey thresholds for mechano-sensitive AM- and C- fibers. Data have been normalized by logarithmic (ln) transformation. Vertical bars represent the geometric mean. Rap, Rapamycin; Veh, vehicle.
(1.81 MB TIF)
