# Supplementary material for: Local Translation in Primary Afferent Fibers Regulates Nociception
Source: PLoS One. 2008 Apr 9;3(4):e1961. doi: 10.1371/journal.pone.0001961 (PMC2276314; doi:10.1371/journal.pone.0001961)
Supplement: Text S1 — Acute nociceptive thresholds are not influenced by local rapamycin injections (0.02 MB DOC) [file pone.0001961.s001.doc]

# Text S1: Acute nociceptive thresholds are not influenced by local rapamycin injections.

First, we investigated the effect of rapamycin on withdrawal latencies to heat stimulation using the Hargreaves test. For this, animals received a single intraplantar injection of 250µM rapamycin or its vehicle in the center of the surface of the left hindpaw. Withdrawal latencies to heat were measured 30 min before (= baseline), and 1, 2, 4, 6 and 24 h after the injection. Intraplantar injection of rapamycin did not change withdrawal latencies to heat stimulation when compared with vehicle (Fig. 4A).

Second, we studied the effect of intraplantar injection of rapamycin on mechanical withdrawal thresholds with both Von Frey hairs and pinprick stimulation (Fig. 4B and 4C).

*Von Frey hairs:*

When animals received either an injection of rapamycin alone (2.5 µM, N=5 and 250 µM, N=11) or appropriate vehicle (N=16) in the lateral part of the surface of the hindpaw, there was no effect of rapamycin on baseline mechanical thresholds after stimulation of the same lateral part of the plantar surface. Measures were taken 1 h before (= baseline), and 1, 2, 4, 6 and 24 h after the intraplantar injection. However, a 50% mean decrease in mechanical withdrawal threshold was found over 24h in the three groups of animals maybe due to the mild inflammation induced by the vehicle (time effect: F_5,145_ = 6.72, *P* < 0.001) (Fig. 4B and Fig. S3A).

Data not shown for the low dose of rapamycin (2.5 µM).

## Pin Prick stimulation:

When rats were given intraplantar injections of rapamycin alone (50 µl, 2.5µM, N=8 or 250 µM, N=8) or its appropriate vehicle (N=16) in the lateral part of the surface of the hindpaw there was no difference in withdrawal response duration between the three groups. Withdrawal response durations were measured 1h before and 1, 2, 4, 6 and 24 h after intraplantar injection. However 24 h following the intraplantar injection a small but significant mean increase in withdrawal duration (0.4 ± 0.18s increment from baseline values) could be measured in all animals (time effect: F_5,145_=3.250**,** *P*< 0.05; Fig. 4C), again probably due to the mild inflammation induced by the vehicle.

Data not shown for the low dose of rapamycin (2.5 µM).

## Randall-Siletto test

Finally, rapamycin or vehicle were injected into the dorsal skin of the hindpaw and mechanical withdrawal thresholds where measured before and 4h after using the Randall-Siletto test. There was no effect of rapamycin (or vehicle) on mechanical withdrawal thresholds measured this way. Data no shown.
