# Supplementary material for: Local Translation in Primary Afferent Fibers Regulates Nociception
Source: PLoS One. 2008 Apr 9;3(4):e1961. doi: 10.1371/journal.pone.0001961 (PMC2276314; doi:10.1371/journal.pone.0001961)
Supplement: Text S2 — Rapamycin does not reduce the number of Fos-IR neurons in dorsal horn induced by capsaicin injection in the hindpaw (0.03 MB DOC) [file pone.0001961.s002.doc]

# Text S2: Rapamycin does not reduce the number of Fos-IR neurons in dorsal horn induced by capsaicin injection in the hindpaw.

The effects of rapamycin on the development of central sensitization following capsaicin injection were studied with c-Fos immunohistochemistry. Fos expression has been widely used to map neuronal activity within nociceptive pathways (Bester et al., 1997;Hunt et al., 1987).

Methods:

Animals were divided into 2 groups of 4 rats. Under halothane (2% in oxygen) anesthesia, animals received an injection of rapamycin or vehicle in the center of the left hindpaw. Four hours later after the first injection, all animals received an injection of capsaicin into the same central area of the left hindpaw. Two hours after stimulation, animals were terminally anesthetized and perfused. The spinal cord was removed and 40 µm thick sections from the lumbar cord were cut and stained for c-Fos immunoreactivity (c-Fos-IR).

The c-Fos antibody (1:60,000) was obtained from Calbiochem (La Jolla, CA).

For c-Fos immunohistochemistry, sections were incubated for 1 h in 3% normal goat serum, 0.3% Triton X-100 and 0.6% of hydrogen peroxide in 0.1M PB. Then, sections were incubated overnight in a rabbit polyclonal antibody anti-c-Fos (Ab-5; Calbiochem, La Jolla, CA) diluted in 0.1M PB (1:60,000). Sections were washed in 0.1M PB and incubated for 1 h in a goat anti-rabbit biotinylated antibody (Vector Laboratories, Burlingame, CA; 1:500 in PB). Sections were washed again in 0.1 M PB and incubated for 2 h in avidin-biotin peroxidase complex (ABC Elite; Vectastain, Vector Laboratories). After washes, sections were incubated for 5 min in a solution containing 0.05 % of 3,3' diaminobenzidine (DAB) and 0.2 % ammonium nickel sulfate in H_2_O. Finally, the reaction was stopped by washes in H_2_O and then PB. The sections were mounted on gelatin-coated slides and coverslipped.

## Counting of c-Fos-labeled cells: the superficial dorsal horn was divided into three ipsilateral domains: the superficial laminae I‑II, the laminae III‑IV and the deep laminae V‑VII. For each rat, Fos-immunoreactive (Fos-IR) neurons counted in the 4 most labelled sections were averaged, and the mean was used for further statistical analysis.

Data from the c-Fos study were analysed by multivariate analysis.

Results:

There was no difference between rapamycin and vehicle pretreated animals in capsaicin induced c-Fos-IR neurons in any the three ipsilateral domains studied: the superficial laminae I‑II (57.1 ± 3.7 *vs* 54.8 ± 5.7 for rapamycin and vehicle respectively), the laminae III‑IV (30.1 ± 3.1 *vs* 30.3 ± 4.1) and the deep laminae V‑VII (38.8 ± 5.2 *vs* 46.5 ± 4.8). These results support our contention that capsaicin-responsive fibers, which are mainly C‑ fibers, are not sensitive to rapamycin.

Reference List

1. Bester H, Matsumoto N, Besson JM, Bernard JF (1997) Further evidence for the involvement of the spinoparabrachial pathway in nociceptive processes: a c-Fos study in the rat. J Comp Neurol 383: 439-458.

2. Hunt SP, Pini A, Evan G (1987) Induction of c-fos-like protein in spinal cord neurons following sensory stimulation. Nature 328: 632-634.
