# Supplementary material for: Local Translation in Primary Afferent Fibers Regulates Nociception
Source: PLoS One. 2008 Apr 9;3(4):e1961. doi: 10.1371/journal.pone.0001961 (PMC2276314; doi:10.1371/journal.pone.0001961)
Supplement: Text S3 — SNI surgery (0.02 MB DOC) [file pone.0001961.s003.doc]

# Text S3: SNI surgery

Following spared nerve injury (SNI), rats showed an enhanced response to pinprick stimulation in the lateral part of the hindpaw the sural territory (Decosterd and Woolf, 2000). Baseline withdrawal duration of 0.5 + 0.2 s increased to 9 + 0.4 s six days following surgery. In sham controls, prior to surgery, the mean withdrawal duration was 0.5 + 0.2 s and it remained unchanged at day 6 after the surgical procedure. Statistical analysis revealed a 'time x surgery' interaction demonstrating that sensitivity was increased over time in animals receiving SNI surgery (F_1,24_ = 16.7, *P* < 0.001).

Reference List

1. Decosterd I, Woolf CJ (2000) Spared nerve injury: an animal model of persistent peripheral neuropathic pain. Pain 87: 149-158.
