# Supplementary figures and images for: Local Translation in Primary Afferent Fibers Regulates Nociception
Source: PLoS One. 2008 Apr 9;3(4):e1961. doi: 10.1371/journal.pone.0001961 (PMC2276314; doi:10.1371/journal.pone.0001961)

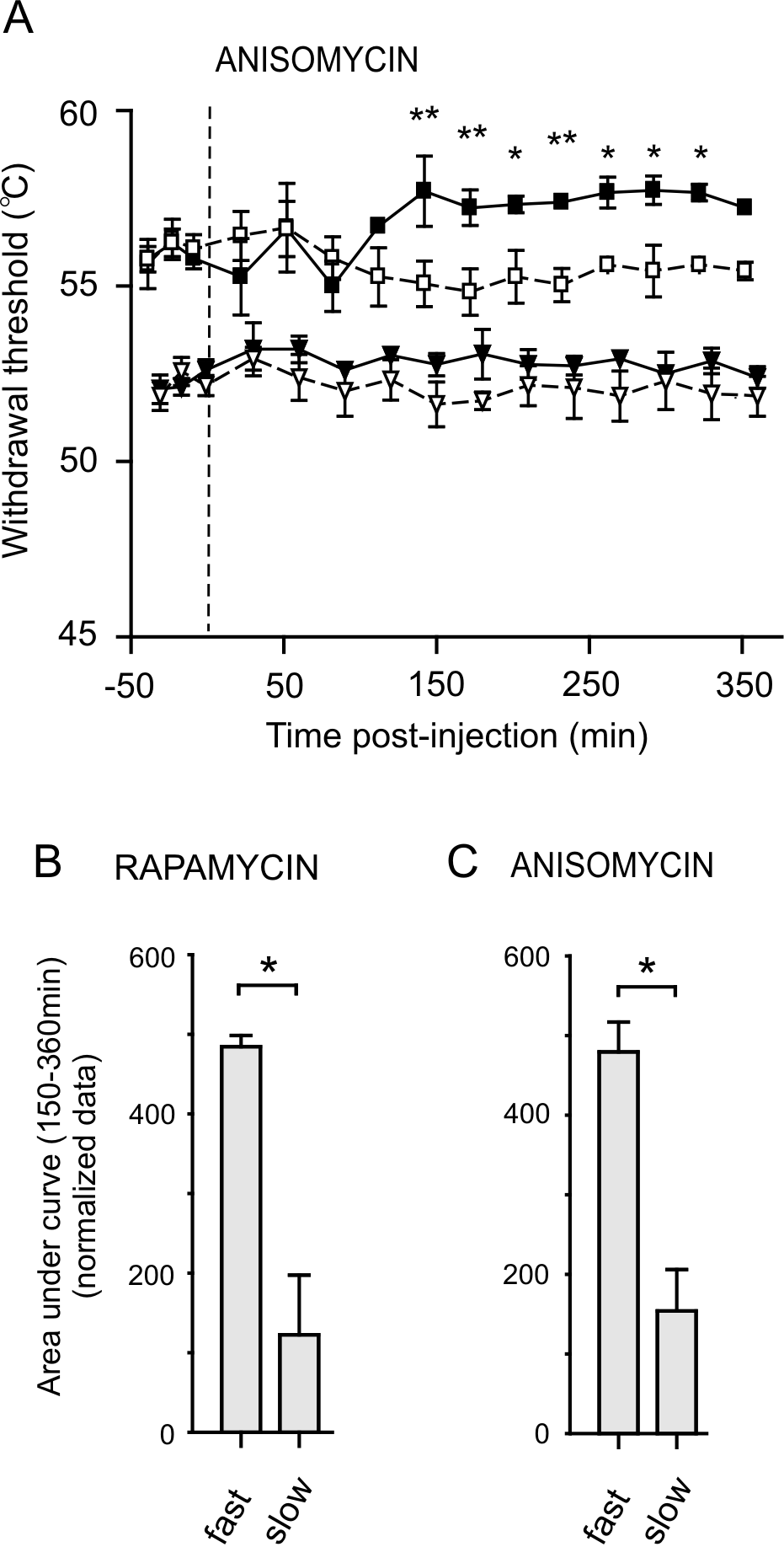

Supplement: Figure S1 — Effects of subcutaneously injected rapamycin or anisomycin on A and C nociceptor-evoked paw withdrawal thresholds. A, Time-course effects of anisomycin (50 µl, 4.7 mM), or vehicle, on paw withdrawal thresholds to fast and slow heat ramps that preferentially activate A- and C-nociceptors respectively. N = 3 in each group. Mean±SEM heat withdrawal threshold ({degree sign}C) for the injected hindpaw is illustrated. Vertical dashed line indicates the drug injection time. B, C, Area under the curve between 150–360 minutes post-injection of rapamycin (B) and anisomycin (C). The data are normalised with respect to the effect of vehicle injection over the same time period; data are expressed as mean±SEM and analysed using student's paired t-test. *, P<0.05; **,P<0.01. (1.71 MB TIF) [file pone.0001961.s004.tif]

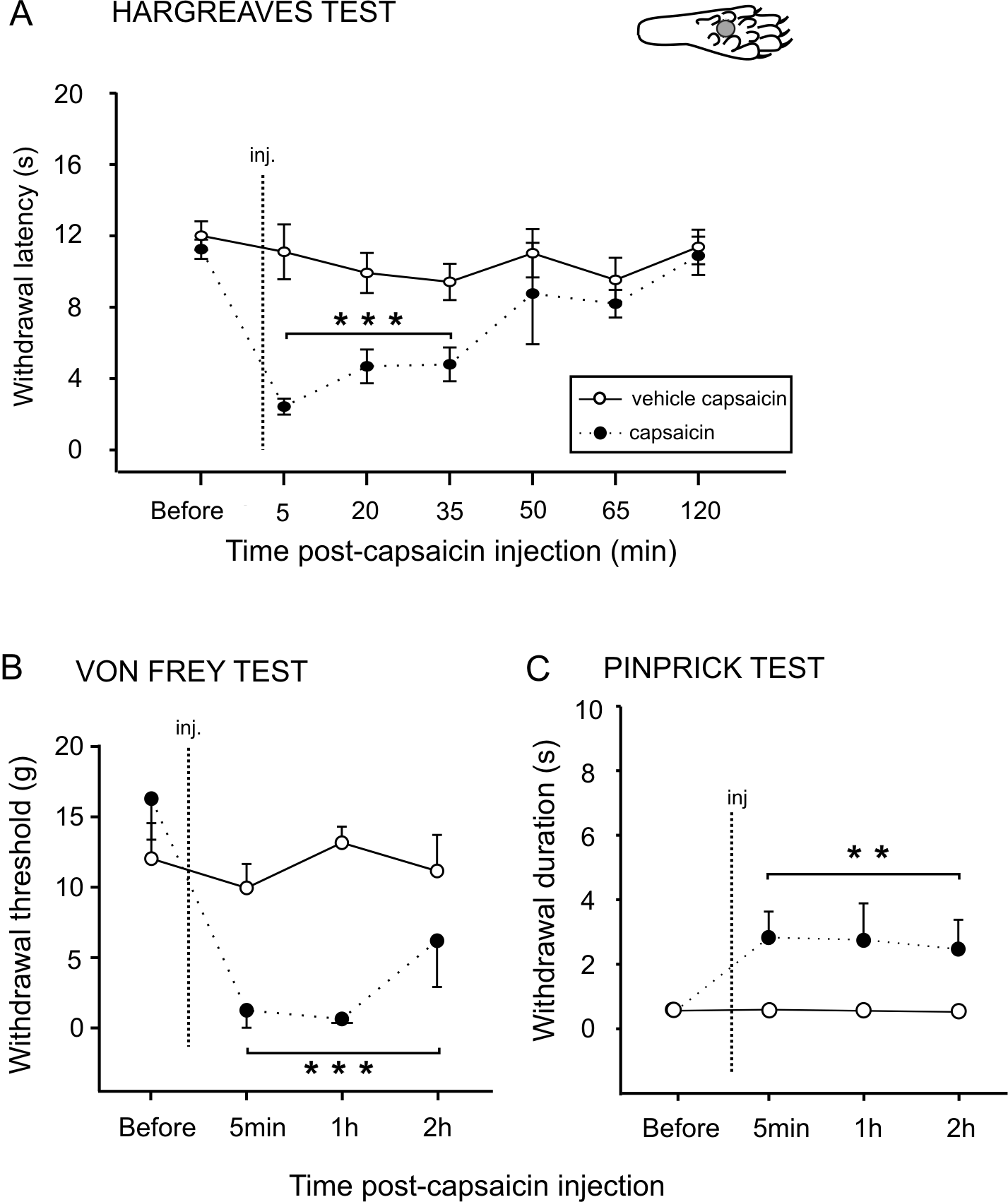

Supplement: Figure S2 — Capsaicin induces local increase in thermal and mechanical sensitivity. Effects of intraplantar injection of 10 µl capsaicin (10 mM) on withdrawal latency to heat, measured in the center of the hindpaw (A), mechanical flexor reflex withdrawal threshold measured in the lateral surface of the paw (B), withdrawal response duration after nociceptive mechanical stimulation (pinprick stimulus) of the lateral plantar surface of the paw (C). Mean±SEM is illustrated in each panel.**, P<0.01; ***,P<0.001. N = 5–6 in each group. (2.43 MB TIF) [file pone.0001961.s005.tif]

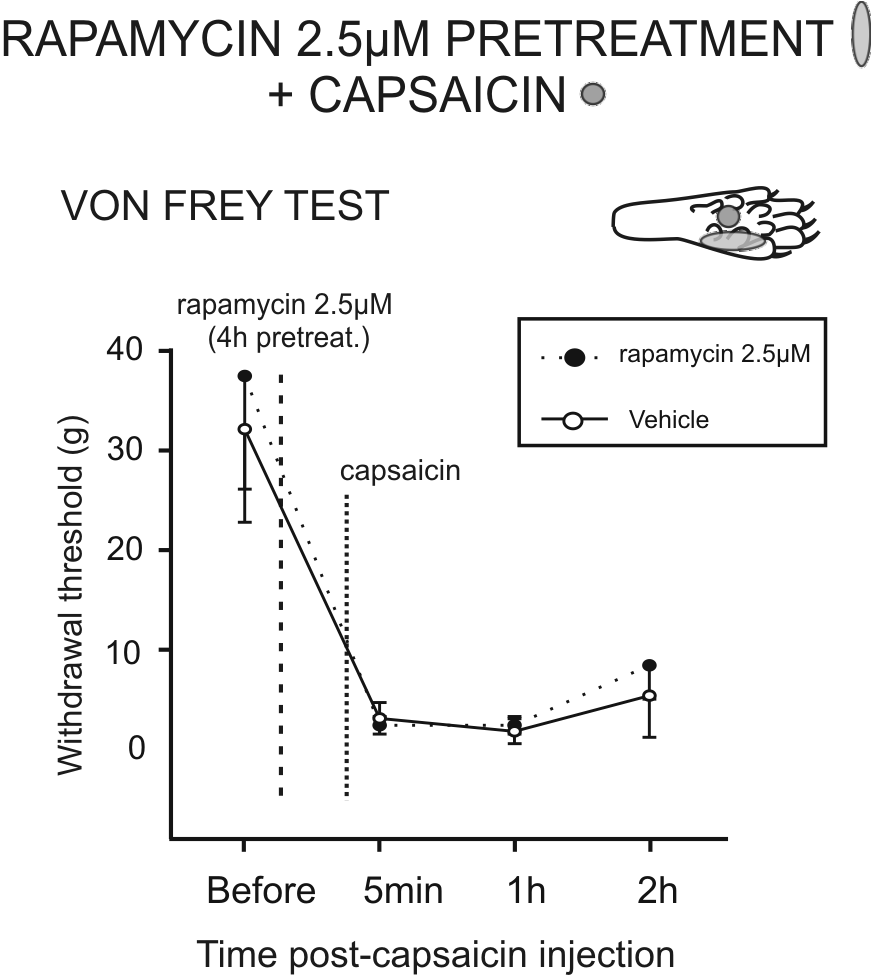

Supplement: Figure S3 — Low dose of rapamycin (2.5 µM) has no effect on capsaicin-induced increased mechanical sensitivity. Secondary mechanical hyperalgesia in lateral plantar hindpaw was generated by injecting capsaicin into the central plantar surface of the hindpaw. Effect of lateral intraplantar injection of rapamycin 2.5 µM or vehicle on mechanical withdrawal threshold measured after injection of capsaicin using Von Frey hairs. N = 6 per group. (0.85 MB TIF) [file pone.0001961.s006.tif]

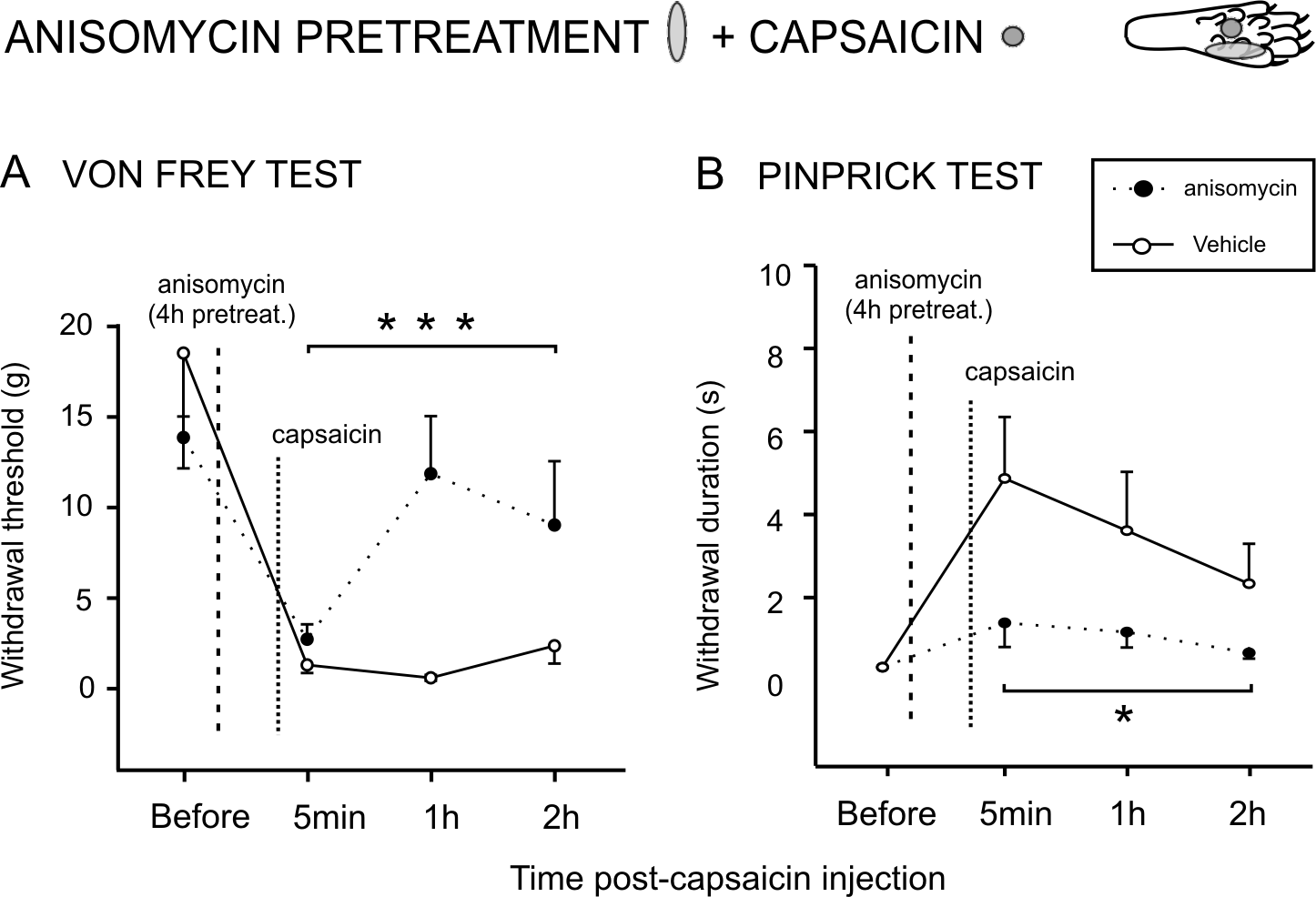

Supplement: Figure S4 — Anisomycin blocks capsaicin-induced secondary mechanical hyperalgesia. Secondary mechanical hyperalgesia in lateral plantar hindpaw was generated by injecting capsaicin into the central plantar surface of the hindpaw. Effect of lateral intraplantar injection of anisomycin (50 µl, 4.7 mM; 4 h before capsaicin) or vehicle on mechanical withdrawal threshold measured after injection of capsaicin using Von Frey hairs (A) and on withdrawal response duration to pinprick stimulation after capsaicin (B). (N = 7–8 for Von Frey and N = 12 for pinprick). (1.43 MB TIF) [file pone.0001961.s007.tif]

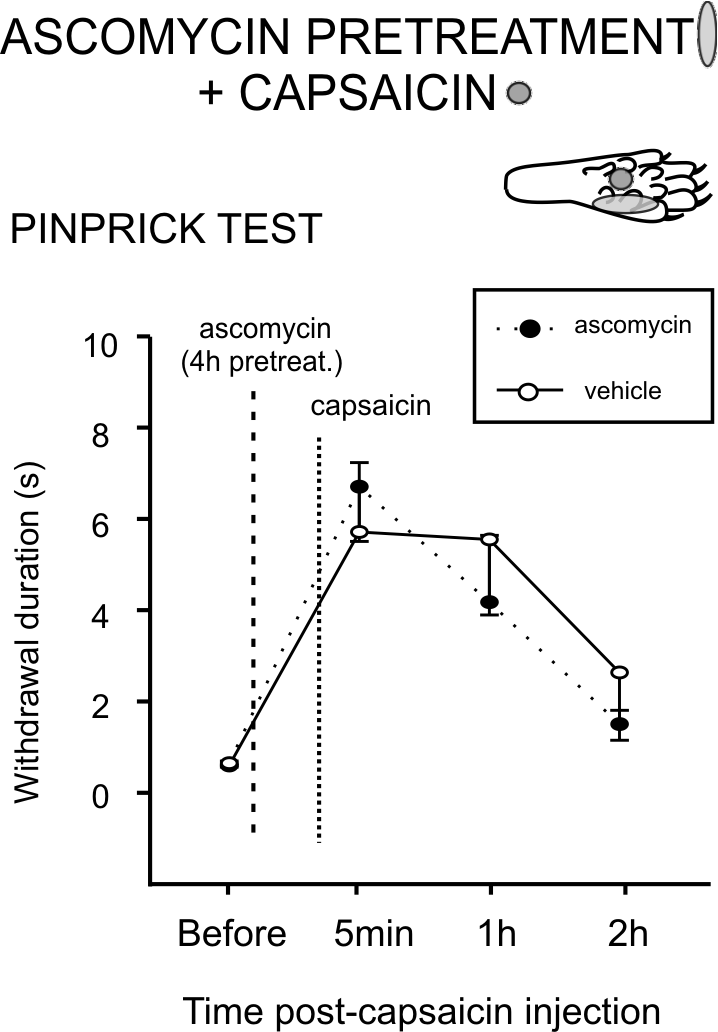

Supplement: Figure S5 — Ascomycin does not change the secondary mechanical hyperalgesia that follows capsaicin injection. Secondary mechanical hyperalgesia in lateral plantar hindpaw was generated by injecting capsaicin into the central plantar surface of the hindpaw. Effect of lateral intraplantar injection of ascomycin or vehicle on withdrawal response duration to pinprick stimulation after capsaicin. (N = 12)M (0.74 MB TIF) [file pone.0001961.s008.tif]

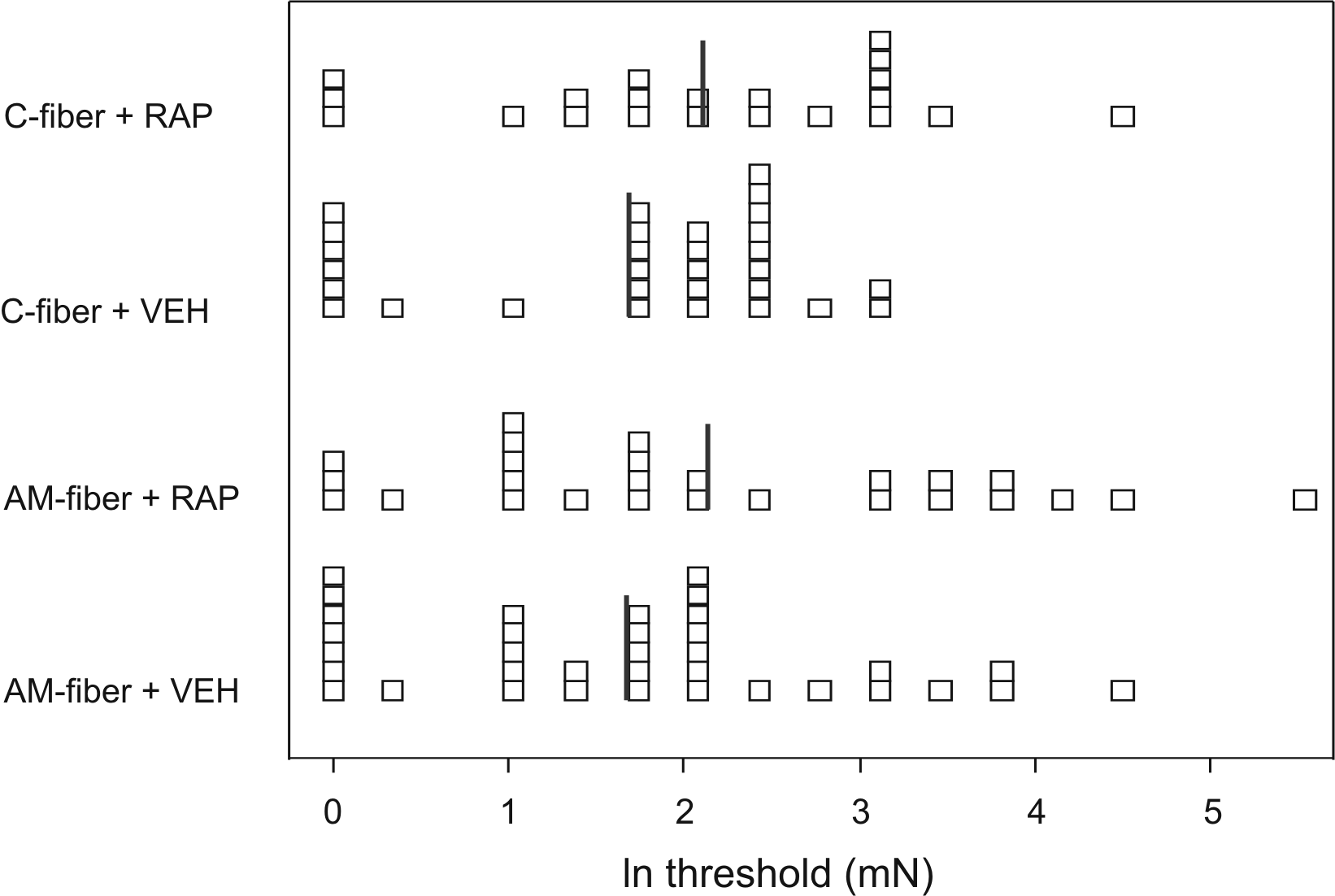

Supplement: Figure S6 — Dotplots showing Von Frey thresholds for mechano-sensitive AM- and C- fibers. Data have been normalized by logarithmic (ln) transformation. Vertical bars represent the geometric mean. Rap, Rapamycin; Veh, vehicle. (1.81 MB TIF) [file pone.0001961.s009.tif]
